# Retinitis pigmentosa is associated with shifts in the gut microbiome

**DOI:** 10.1038/s41598-021-86052-1

**Published:** 2021-03-23

**Authors:** Oksana Kutsyr, Lucía Maestre-Carballa, Mónica Lluesma-Gomez, Manuel Martinez-Garcia, Nicolás Cuenca, Pedro Lax

**Affiliations:** 1 Department of Physiology, Genetics and Microbiology, University of Alicante, Alicante, Spain; 2 Institute Ramón Margalef, University of Alicante, Alicante, Spain

**Keywords:** Retinal diseases, Microbiome, Neurodegeneration

## Abstract

The gut microbiome is known to influence the pathogenesis and progression of neurodegenerative diseases. However, there has been relatively little focus upon the implications of the gut microbiome in retinal diseases such as retinitis pigmentosa (RP). Here, we investigated changes in gut microbiome composition linked to RP, by assessing both retinal degeneration and gut microbiome in the rd10 mouse model of RP as compared to control C57BL/6J mice. In rd10 mice, retinal responsiveness to flashlight stimuli and visual acuity were deteriorated with respect to observed in age-matched control mice. This functional decline in dystrophic animals was accompanied by photoreceptor loss, morphologic anomalies in photoreceptor cells and retinal reactive gliosis. Furthermore, 16S rRNA gene amplicon sequencing data showed a microbial gut dysbiosis with differences in alpha and beta diversity at the genera, species and amplicon sequence variants (ASV) levels between dystrophic and control mice. Remarkably, four fairly common ASV in healthy gut microbiome belonging to *Rikenella spp., Muribaculaceace* spp., *Prevotellaceae* UCG-001 spp., and *Bacilli* spp. were absent in the gut microbiome of retinal disease mice, while *Bacteroides caecimuris* was significantly enriched in mice with RP. The results indicate that retinal degenerative changes in RP are linked to relevant gut microbiome changes. The findings suggest that microbiome shifting could be considered as potential biomarker and therapeutic target for retinal degenerative diseases.

## Introduction

Neuronal degeneration is an intricate process in which intrinsic and environmental stress can affect vulnerable neurons to promote disease. Mounting evidence highlights the importance of a bidirectional crosstalk between the gastrointestinal bacteria and the central nervous system^1–3^, and the impact of gut microbiome on brain and behavior is being extensively reported in the literature^4–8^. The homeostasis of the gut microbiome is critical for maintaining human health, and imbalances in the microbial composition of the gut profoundly influences critical features of host physiology, including the development of metabolic disorders such as diabetes and obesity^7,9^. Emerging data support the potential role for the gut microbiome in modulating many aspects of the brain function and behavior, with effects on the stress response, mood and anxiety disorders, motor activity, social interaction and memory, among others^10–15^.

The interplay between the brain and the gut bacteria is mainly mediated by neural and immune networks, with crosstalk interactions between both systems^3,16^. Thereby, the microbiome–gut–brain signaling system influences key brain processes, including neurogenesis, neurotransmission, neuroinflammation and neuronal degeneration^17–20^. In this context, experimental data has proved that intestinal microbiome influences brain response to injury^21–23^, and vice versa^24^, so that changes in gut microbiome may affect recovery and treatment following brain damage^24^. Besides, dysbiosis of the human gut microbiome has been associated with neurodegenerative disorders of the central nervous system that include Parkinson’s, Alzheimer’s and Huntington’s disease^25–29^.

The retina has been historically considered a window to the brain, and anatomically the retina can be regarded as an extension of the central nervous system The structural and functional features of the retina make this tissue highly vulnerable to stressors, and homeostasis alterations significantly influence the progress of retinal pathologies^30^. Moreover, the retina reflects some of the pathological alterations of many neurodegenerative diseases and may provide information of brain pathology severity^31,32^. In this context, a few recent studies have linked gut microbiome changes with some retinal degenerative diseases^33,34^, including age-related macular degeneration (AMD)^35–39^, glaucoma^40–43^ and diabetic retinopathy^44^, even though the published results vary depending on the type and stage of the disease and between studies. On the other hand, in a previous study we have demonstrated that invasive infection from gastrointestinal microbiome can induce activation of retinal microglia^45^, the primary resident immune cell of the retina.

Retinitis pigmentosa (RP) is a heterogeneous group of inherited diseases that cause photoreceptor degeneration, eventually leading to complete blindness^30^. The death of photoreceptors is accompanied by chronic microglial activation and neuroinflammatory processes^46–48^, concomitant with an increase of reactive oxygen species^49–52^. RP disease-causing mutations have been identified in more than 80 different genes^53^. The rd10 mouse model of RP has a missense mutation in the phosphodiesterase 6b (*Pde6b*) gene^54,55^, inducing rod photoreceptor degeneration, which leads to secondary cone photoreceptor death^30,56^. Time courses of photoreceptor cell death and subsequent retinal degeneration in rd10 mice closely resembles the human disease process^30,57^.

To date, there are no empirical studies in the literature analyzing the gut microbiome composition in retinitis pigmentosa. In this study we analyzed the gut microbiome in control and rd10 mice at postnatal day (P) 32, when dystrophic animals are expected to have suffered from extensive retinal degeneration. We assessed retinal degeneration by functional electroretinography (ERG) and morphological techniques, and we evaluated the gut microbiome by Illumina 16S rRNA gene amplicon sequencing. We have confirmed degenerative changes in neuronal and glial retinal cells and demonstrated alterations in gut microbiome populations of RP animals. These results reinforce the general concept of the interdependence of gut microbiome and the central nervous system homeostasis and suggest that gut microbiome could potentially constitute a therapeutic target for RP and other retinal degenerative diseases.

## Results

### Degenerative changes in retinitis pigmentosa mice

**Figure 1:**
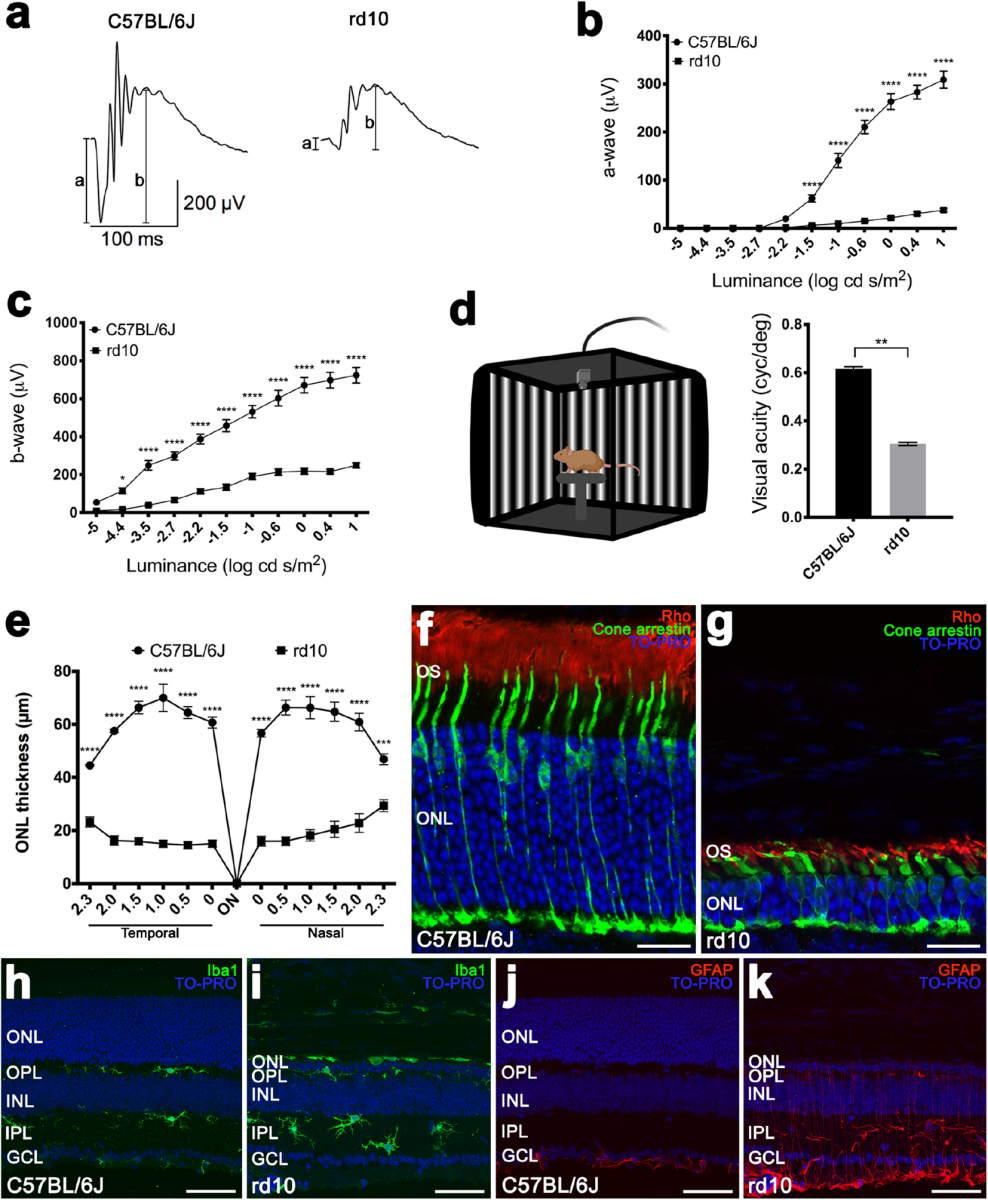
Retinal changes in RP mice. (**a**) Scotopic ERG responses to 1 log cd s/m^2^ flashes from a normal C57BL/6J (left) and dystrophic rd10 (right) mouse. The amplitudes of both the a- and b-waves are represented. (**b, c**) Luminance-response curves for the a- (**b**) and b-(**c**) waves of C57BL/6J (circles) and rd10 mice (squares). (**d**) Configuration of the optomotor system (left, image created using BioRender; https://biorender.com/) and visual acuity thresholds for C57BL/6J and rd10 mice (right). (**e**) Mean outer nuclear layer thickness in C57BL/6J (circles) and rd10 (squares) mice, quantified in both the temporal and the nasal side of the retina. (**f, g**) Retinal sections showing the outer retina of a C57BL/6J (**f**) and rd10 (**g**) mouse immunolabeled against cone arrestin (cone cells, in green) and rhodopsin (Rho, rod cells, in red). Nuclei were stained with TO-PRO 3 (in blue). (**h–k**) Retinal sections from a C57BL/6J (**h, j**) and rd10 (**i, k**) mouse, immunolabeled against Iba1 (microglia, in green) or GFAP (activated macroglia, in red). The cell nuclei were stained with TO-PRO 3 (in blue). ANOVA, Bonferroni’s test: *p < 0.05, **p < 0.01, ***p < 0.001, ****p < 0.0001. ON: optic nerve, OS: outer segments, ONL: outer nuclear layer, OPL: outer plexiform layer, INL: inner nuclear layer, IPL: inner plexiform layer, GCL: ganglion cell layer. Scale bars: (**f, g**), 20 µm; (**h–k)**, 50 µm.

RP mice showed altered retinal function and morphology. ERG flash responses from rd10 mice were smaller than those obtained in C57BL/6J mice (Fig. 1a). In rd10 mice, maximum amplitudes observed for scotopic a- and b-waves were 12% and 34% (respectively) of the values obtained in C57BL/6J mice (Fig. 1b,c). Also, visual acuity showed visual thresholds significantly smaller in rd10 mice (50% less) than those obtained in control mice (Fig. 1d). On the other hand, the mean thickness of the ONL was smaller in rd10 than in control mice throughout the retina (Fig. 1e). On average, the ONL thickness in rd10 mice was 31% of the values obtained in C57BL/6J mice (18.6 ± 1.6 vs. 60.4 ± 2.0 µm). Cone photoreceptors in control mice showed a normal morphology with visible inner and outer segments and long axons, and normal pedicles (Fig. 1f). Conversely, in rd10 mice cones exhibited a degenerated morphology, with small size cones and an almost absent inner and outer segments (Fig. 1g). In these animals, cone axons were almost loss and pedicles came out from the cell bodies. Besides, rod outer segments of RP mice were shorter and more disorganized than those of control animals (Fig. 1f,g).

Photoreceptor death was associated to reactive gliosis in the retina of rd10 mice. In control mice, Iba1-positive microglial cells were scarce in the outer retina and exhibited morphological features typical of resting microglia (Fig. 1h). By contrast, rd10 mice showed evident changes in Iba1-positive cells, with higher number of positive cells than observed in C57BL/6J retinas, and abundant iba1-positive cells in the outer nuclear layer (Fig. 1i). Moreover, Iba1-positive cells in rd10 retinas showed a phenotype characteristic of reactive microglia (Fig. 1i). Immunoreactivity for glial fibrillary acidic protein (GFAP) also evidenced a reactive gliosis in rd10 retinas. In C57BL/6J retinas, GFAP immunoreactivity was present only in the inner margin of the retina, corresponding to astrocyte cells (Fig. 1j). By contrast, retinal GFAP immunoreactivity in rd10 was present not only in the inner margin of the retina but also throughout Müller cells (Fig. 1k), which points to the activation of macroglial cells.

### General gut microbial composition features

**Figure 2:**
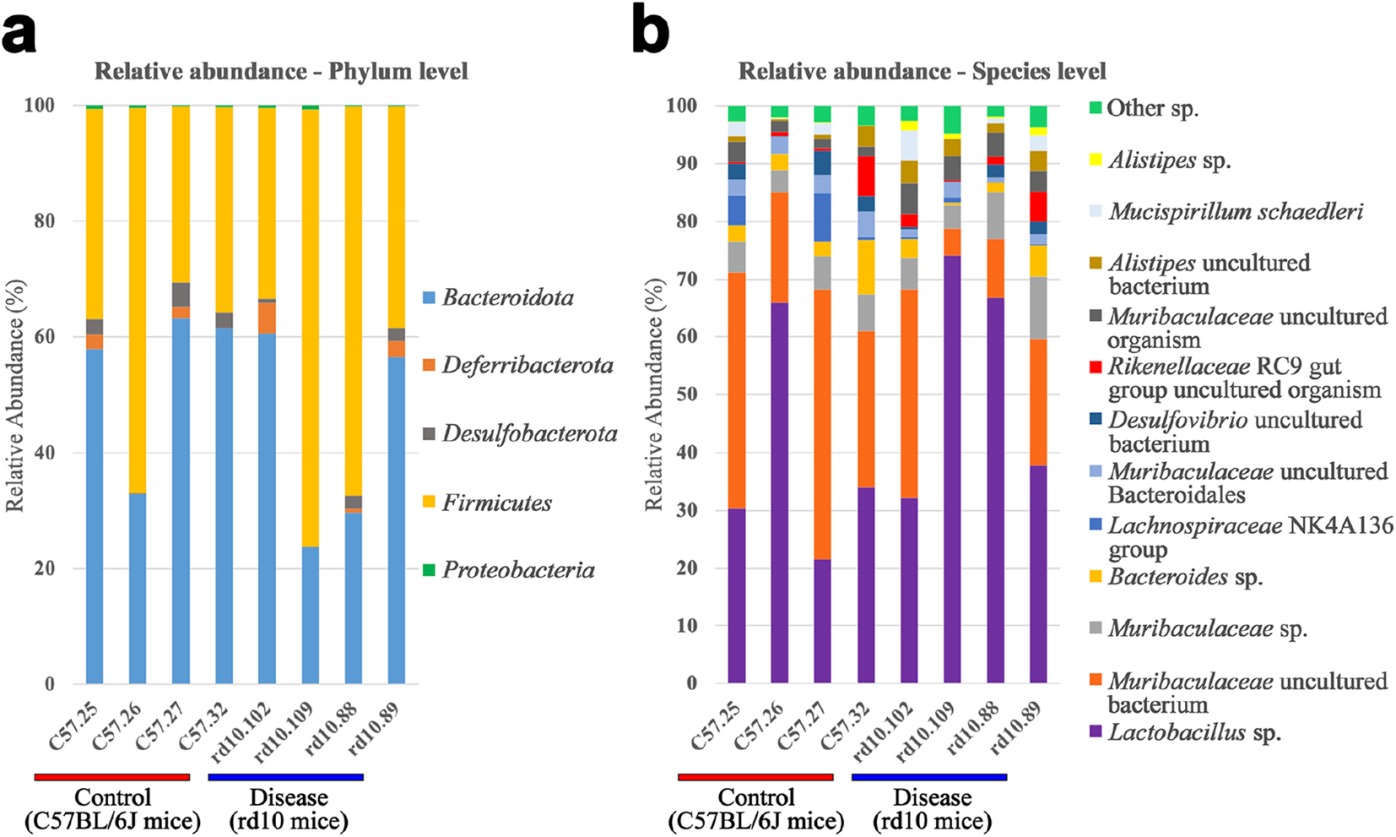
Taxonomic analysis of the mouse gut microbiome. (**a**) Relative abundance (%) at Phylum level for C57BL/J6 and rd10 mice gut microbiota. Dominant phyla were *Bacteroidota* and *Firmicutes* in both groups. (**b**) Relative abundance (%) of the most abundant species found in C57BL/6J and rd10 samples. Only species which had at least 1% of relative abundance in one of the samples were represented.

DNA from 8 mice’s gut and stool (4 from C57BL/6J mice and 4 from rd10 mice) was extracted and the 16S rRNA marker gene was amplified with PCR using the primers 341F and 805R, and then sequenced with Illumina technology. Reads were quality-filtered, merged (see methods and Supplementary Table S1) and analyzed with QIIME2.2020^58^. The denoiser tool Deblur, available in QIIME2^58^, was used to remove sequencing errors (57–66% sequences per sample, Supplementary Table S2). After quality control processing, as a mean,  ≈ 100,000 final joined reads were obtained for denoising analysis that delivered ≈ 38,000 reads for taxonomic classification and 16S rRNA gene data analysis (Supplementary Tables S1 and S2). Regarding general taxonomic features (Supplementary Figure S1), in both healthy C57BL/6J mice and diseased rd10 mice, the phyla *Bacteroidota* and *Firmicutes* were predominant in the gut representing 96% of the relative microbial abundance, followed by *Deferribacterota* and *Desulfobacterota* (Fig. 2a). At the species level, 12 were predominant in both mice groups and represented from 94.23 up to 97.91% of the relative abundance per sample (Fig. 2b). *Lactobacillus* spp. was the most abundant specie in both rd10 (≈ 53%) and C57BL/6J (≈ 38%) mice while an uncultured *Muribaculaceae* bacterium was placed the second most abundant specie (Fig. 2b). Other common bacterial species in the gut were also detected, such as *Bacteroides spp. and Alistipes spp.*

### Altered gut microbiome: differences in alpha and beta diversity

**Figure 3:**
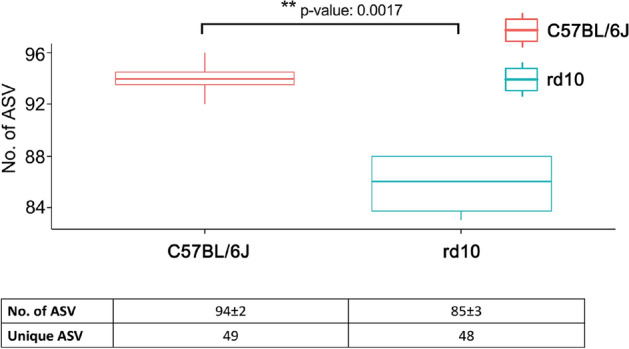
Amplicon sequence variant analysis. Top panel: boxplot comparing the number of amplicon sequence variants (No. of ASV). One-way ANOVA test showed statistically significant differences between the number of ASV in healthy (red) and rd10 (blue) mice. First row of the table contains mean ± SD of ASV, the second one contains the number of unique ASV (not shared with the other group) for healthy (left) and rd10 mice (right). Graphic performed with the library ggboxplot available in R.

**Figure 4:**
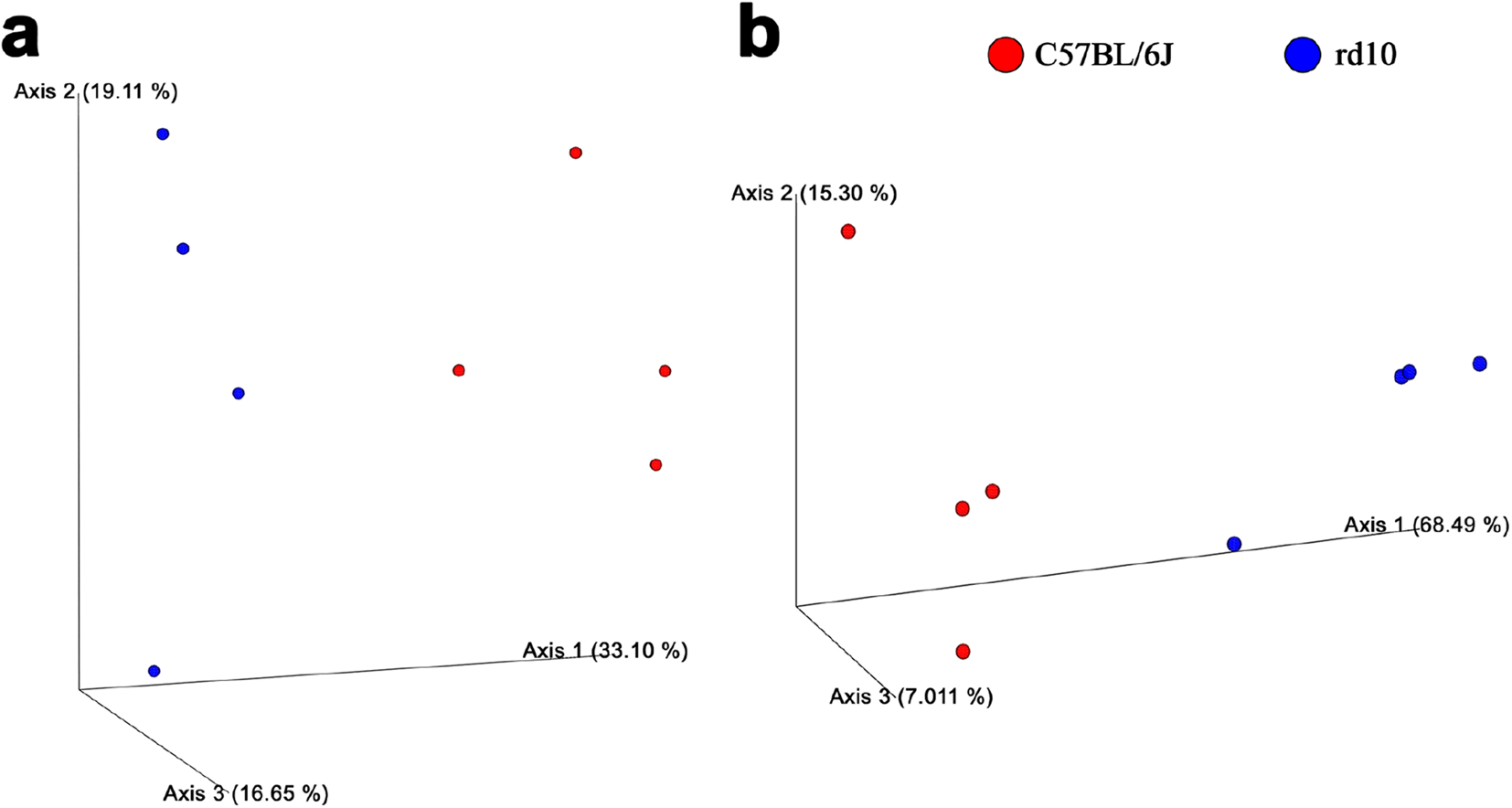
PCoA representation. (**a**) Principal coordinate analysis (PCoA) at species level, where C57BL/6J (red) and rd10 (blue) groups could be differentiated. (**b**) PCoA representing unweighted UniFrac distance for C57/6J (red) and rd10 (blue) mice gut were the two groups are separated from each other. The PERMANOVA test performed showed significant differences between the two groups.

**Figure 5:**
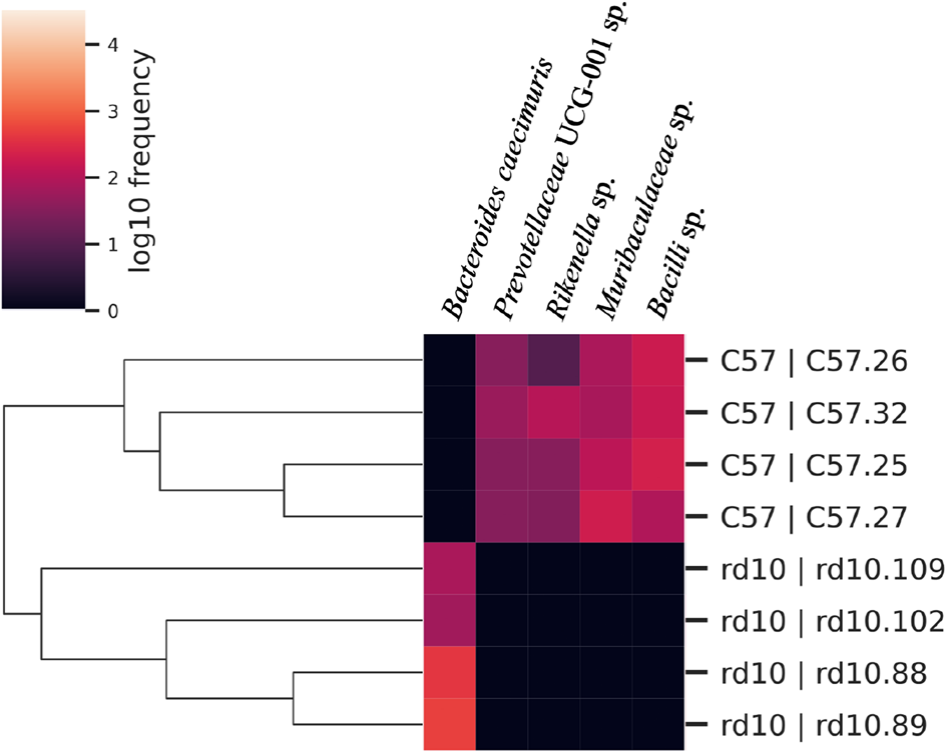
Heatmap that shows species that were identified by ANCOM as more abundant. *Bacteroides caecimuris* was more abundant in rd10 mice, while *Prevotellaceae* UCG-001 spp., *Rikenella* spp., *Muribaculaceae* spp. and *Bacilli* spp. were more frequent in C57BL/6J (C57) compared with the other mice group.

Despite these similarities on general microbial features, apparent alpha and beta diversity differences were found in the microbial gut composition between healthy and diseased mice. First, regarding richness of amplicon sequence variants (ASV), higher number of ASV were found for control mice group (n = 94 ± 2) compared to diseased rd10 mice (n = 86 ± 3) (*p* = 0.0017, Fig. 3). In addition, 49 unique ASV were only found in healthy mice (representing an accumulative relative abundance of 26.7%) whereas 48 were only found in rd10 mice (17.6% of the relative abundance) (see details in Supplementary Table S3). Second, more alpha-diversity was obtained for C57BL/6J healthy control mice when measured with Pielou’s Evenness, Shannon’s Diversity and Faith’s Phylogenetic Diversity indices (Supplementary Figure S1 and Supplementary Table S4). Furthermore, the principal coordinate analysis (PCoA) for the beta-diversity at different taxonomic ranks from family to species (Fig. 4a) and ASV (Fig. 4b) levels showed that C57BL/6J control mice grouped together and separately from rd10 disease mice. Indeed, these beta-diversity differences based on unweighted UniFrac distance were statistically significant (PERMANOVA test, *p* = 0.03, Supplementary Table S4) at the ASV level (Fig. 4b) between control and disease mice using Jaccard (*p* = 0.03) and Bray-courtis (*p* = 0.027) distance indices (Supplementary Table S4). Remarkably, when analyzed those taxa significantly enriched in control and disease mice with ANCOM^59^, which compares the relative abundance of each taxon with all the remaining features of the same category, data showed that four species (*Rikenella spp, Muribaculaceace* spp., *Prevotellaceae* UCG-001 spp., and *Bacilli* spp.) commonly present up to nearly 1% in healthy gut microbiome were absent in rd10 disease mice (Fig. 5 and Supplementary Tables S3 and S5). On the other hand, *Bacteroides caecimuris* was significantly overrepresented in rd10 mice with an average relative abundance of 0.7% (Fig. 5 and Supplementary Tables S3 and S5), while lacking in healthy gut mice. Finally, no difference in microbial composition was found between female versus male mice from both analyzed healthy and disease groups (tested with PERMANOVA, *p* > 0.05, Supplementary Table S4).

## Discussion

Previous studies have linked gut microbiome changes with retinal degenerative diseases. Here we demonstrate for the first time that degenerative changes in neuronal and glial retinal cells concur with shifts in gut microbiome composition in an animal model of retinitis pigmentosa. The reported deteriorations in retinal responsiveness and in photoreceptor cell number and morphology of rd10 mice agrees with that previously shown for these animals^51,52^. Also, retinal reactive gliosis observed in the dystrophic animals are consistent with the increases in microglial cell numbers and Müller cell reactivity described in previous studies^51,52^, and point to the activation of pro-inflammatory pathways in these animals. In this context, previous results have demonstrated significant increases of inflammation markers in rd10 mice^52^, and augmented expression of proinflammatory cytokines has been previously reported by us in RP animals^48^. The inflammatory state in retinitis pigmentosa animals persists throughout the life span even after photoreceptor loss^47^, and concurs with significant increase of oxidative stress^52^. In fact, it is assumed that apoptotic cell removal, inflammation and oxidative stress are common features in all retinal neurodegenerative diseases, including age-related macular degeneration, glaucoma, diabetic retinopathy and retinitis pigmentosa^30^.

Initiation and progression of some prevalent retinal neurodegenerative diseases have also been linked to changes in the homeostasis of gut microbiome^33,34^. In our results, sequencing analysis of the gut microbiome in dystrophic and control mice showed differences in alpha and beta diversity and interestingly, these differences were statistically supported at the ASV level. In recent reviews on best practices for analyzing microbiomes^60,61^, ASV methods have been proposed as the reference metric to unveil differences in terms of microbial composition and have demonstrated sensitivity and specificity as good or better than previous methods and better discriminate ecological patterns^62–65^. Remarkably, there were a large fraction of unique ASV present in only one of the groups (diseased or healthy), which overall contribution in relative microbial abundance variates between 17.6% (diseased mice) and 26.6% (healthy mice). For instance, four ASV classified as *Rikenella spp., Muribaculaceace* spp., *Prevotellaceae* UCG-001 spp., and *Bacilli* spp. were common in healthy gut microbiome but absent in the gut microbiome of retinal disease mice. Oppositely, *B. caecimuris,* normally rare in healthy gut microbiome, was significantly abundant in diseased mice. Thus, data showed a taxonomic partitioning for several ASV in the gut microbiome of diseased and healthy gut microbiomes. Precisely, these striking differences in terms of presence *vs*. absence of these unique ASVs likely explain our results on ASV microbial composition (Fig. 3) based on unweighted beta-diversity model (i.e. low and high abundant ASV have the same importance)^66^. When analyzing the data based on weighted beta diversity metric, which takes into account the relative abundance of all ASVs, differences were not statistically significant between diseased and healthy mice. This might be explained because with weighted beta diversity model, the relative contribution of most predominant species and ASVs, such as *Lactobacillus* and other abundant species described in Fig. 2b, likely mask the overall contributions of those less abundant species/ASV, which individually have a minor contribution with a relative abundance between 0.06 and 6.85% each one depending on the group, despite there were contrasting differences in absence or presence for several ASV taxa (Fig. 5). Those unique low abundant ASV representing different rare bacterial taxa could be important in the gut’s ecosystem since it has been proved that rare or low frequent bacteria have key roles driving ecosystems^67^, for instance, determining the bacterial gut composition in termite after different diet variations^68^.

It has been reported that genera *Rikenella* and *Prevotella* were prevalent in 101 healthy mice gut microbiomes (including the C57BL/6J strain), being identified in 73.3% and 79.2% of the analyzed samples, thus being considered part of the healthy core of mice gut^69^. In addition, bacteria belonging to the family *Muribaculaceae* are related to colonic inner mucus layer formation and barrier function^70^, and the abundance of *Muribaculaceae* correlates with increased production of short-chain fatty acids and enhanced longevity in mice^71^. Besides, it has been demonstrated that relative abundance of *Muribaculaceae* negatively correlates with inflammatory mediators^70,72^, and that fecal short-chain fatty acids concentrations are significantly reduced in Parkinson disease patients compared to controls^73^. On the other hand, the abundance of *Prevotellaceae* has been reported to be reduced in feces of patients with neurological and psychiatric disorders^16^, including multiple sclerosis^74^, Parkinson disease^25,73^ or major depressive disorder^75^. Furthermore, previous studies have proved that the presence in gut microbiome of *Bacilli* spp., as *Lactobacillus*, can contribute to the production of short-chain fatty acids and collaborate in the maintenance of immune cells and the production anti-inflammatory response^76,77^. Therefore, we can infer that the decline in the population density of these bacterial species may be related to the inflammatory and degenerative processes in RP mice.

Several different mechanisms have been proposed to explain how changes in the gut microbiome are linked to ocular diseases^34^. Microbial imbalance can result in disruptions of the intestinal permeability and the blood-retinal barrier^43^, thus allowing bacteria and their products to induce ocular cells to an inflammatory state^34,35^. Moreover, it has been hypothesized that gut dysbiosis may be a cause of increased levels of oxidative stress in the central nervous system^78^. But also vice versa, central nervous system injuries may cause changes in the gut environment, and trigger alterations of gut microbiome^79^. In this context, it has been demonstrated that brain injury may induce changes in the gut microbiome composition via altered autonomic balance^24^. All these hypotheses are in concordance with the context of neuroinflammation, oxidative stress and cell death observed in RP mice. The link between gut microbiome composition and retinal health suggests that different stages of retinal degeneration might correspond to different gut microbiome changes. In this context, it has been demonstrated that human pharyngeal microbiome varies depending on the stage of the disease in age-related macular degeneration^80^. On the other hand, in our opinion, the restoration of the gut microbiome could prevent or reverse retinal degeneration. Previous studies have demonstrated that modification of the gut microbiota by microbiota transplantation^81^, or by changing the diet^36^, can attenuate the development of age-related macular degeneration, and that restructuring of the gut microbiome by intermittent fasting prevents retinopathy in diabetic mice^82^.

## Conclusions

Our results confirm previously described alterations in the morphology and function of the rd10 mouse, an animal model of retinitis pigmentosa, and demonstrate for the first time that retinal degenerative changes in neuronal and glial cells occurring in retinitis pigmentosa are concomitant with relevant gut microbiome changes. The findings could be extrapolated to patients suffering from retinitis pigmentosa or other ocular degenerative diseases and suggest that microbiome shifting could be considered as potential biomarker and therapeutic target for human retinal degenerative diseases. We realize that our results are preliminary and hope that it will lead and trigger further studies to elucidate the specificity of the interactions between the gut microbiome and retinitis pigmentosa or other retinal diseases. Continued investigations of the gut-retina axis could reveal unknown aspects of retinal diseases and potentially identify new relevant targets for therapeutic strategies.

## Methods

### Animals

Mice homozygous for the rd10 mutation (B6.CXBI-Pde6brd10/J) (n = 8) and wild-type C57BL/6J mice (Harlan Laboratories, Barcelona, Spain) (n = 8), half male, half female, were used in the study. Animals were maintained in cages under controlled temperature (23 ± 1 °C), humidity (60%) and photoperiod (12 h light/12 h dark, 50 lx). Water and food were provided ad libitum. At the end of the study, animals were humanely sacrificed by a lethal dose of sodium pentobarbital. The study has been approved by the Ethics Committee of the University of Alicante (UA-2018–07-06). All procedures were performed in conformity with current guidelines and regulations on the use of laboratory animals (European Directive 2010/63/EU, NIH, ARVO and ARRIVE) in an effort to reduce the number of animals used and limit unnecessary animal suffering.

### Electroretinographic records

In the morning of postnatal day 32, scotopic ERG responses were recorded bilaterally following previously reported methodology^52^. After overnight dark adaptation, animals were anesthetized under dim red light by intraperitoneal administration of 100 mg/kg of ketamine (Imalgene, Merial Laboratorios S.A., Barcelona, Spain) and 4 mg/kg of xylazine (Xilagesic 2%, Laboratorios Calier, Barcelona, Spain), pupils were dilated with tropicamide 1% (Alcon Cusí, Barcelona, Spain), and the eyes were instilled with 0.2% polyacrylic acid carbomer (Novartis, Barcelona) to reduce dehydration and improve electrical connectivity with the recording electrodes (DTL fiber; Sauquoit Industries, Scranton, PA, USA). A reference needle electrode was placed in the head, under the scalp, and a ground electrode was placed in the mouth. During the recordings, into a Faraday cage, stable body temperature (37 ± 0.3 °C) and absolute darkness was maintained. Light stimuli (10-ms duration) were presented for at 11 logarithmically increasing luminance (from -5 to 1 log cd s/m^2^) by a Ganzfeld led stimulator. The responses to 3 to 10 consecutive stimuli were averaged for each light intensity. The spacing between flashes was 10 s for dim flashes (-5 to -0.8 log cd s/m^2^) and 20 s for bright flashes (0 to 1 log cd s/m^2^). A data acquisition board (DAM50; World Precision Instruments, Aston, UK) was used to amplify and band-pass filter the signal (1–1000 Hz, without notch filtering). Stimuli administration and data acquisition (4 kHz) were accomplished using PowerLab-AD system (AD Instruments, Oxfordshire, UK).

### Optomotor test

Visual acuity (VA) was assessed in C57BL/6J and rd10 mice, by evaluating optomotor responses in the Argos system (Instead, Elche, Spain). As described previously^52^, spatial frequency thresholds were obtained by analyzing the response of the animals to vertically oriented drifting gratings (Fig. 1d). The initial spatial frequency tested was 0.088 cyc/deg and the temporal frequency was 0.8 Hz.

### Tissue and stool collection

After ERG recording, animals were sacrificed, and tissue samples were collected. For microbial analysis, colon and ileum segments were removed and stored at − 80 °C after quick immersion in liquid nitrogen. For morphological analysis of the retinas, the eyes were enucleated after the placement of a suture to mark the dorsal margin of the limbus. The eyes were then fixed with 4% (w/v) paraformaldehyde for 1 h at room temperature, washed with 0.1 M phosphate buffer (PB, pH 7.4) and cryoprotected through a series of increasing concentrations of sucrose (15, 20 and 30% (w/v)). Following, the cornea, lens and vitreous body were gently removed, the eyecups were embedded in Tissue-Tek OCT (Sakura Finetek, Zoeterwouden, Netherlands), frozen with liquid nitrogen and cut with a cryostat (CM 1900, Leica Microsystems, Wetzlar, Germany). Sections of thickness 16 μm were mounted on glass slides (Superfrost Plus; Menzel GmbH and Co. KG, Braunschweig, Germany) and stored at − 20 °C.

### DNA extraction

For the microbiome study, 8 tissue and stool samples were used. Half of them were rd10 and the other half were C57BL/6J, also there were 2 males and 2 females in each group. DNA was extracted from the samples using DNAeasy PowerSoil Pro (QIAGEN, Germany) according to the manufacturer’s protocol, including an extra sample incubation with CD2 at 4 °C during 5 min before being centrifuged. All centrifugations were carried at 15,100 G, minus the one used for removing the residual solution C5, centrifuged at 16,100 G.

### PCR and sequencing of 16S rRNA gene amplicons

DNA from fecal and colon samples was subjected to amplification of polymerase chain reaction (PCR) using Pro341F (5-’TCGTCGGCAGCGTCAGATGTGTATAAGAGACAGCCTACGGGNBGCASCAG3′) and Pro805R (5′GTCTCGTGGGCTCGGAGATGTGTATAAGAGACAGGACTACNVGGGTATCTAATC-3′), targeting the V3-V4 region of 16S rRNA gene. The PCR conditions were: 94 °C for 3 min, 25 cycles of 94 °C for 45 s, 51 °C for 1 min and 72 °C for 10 min. This was followed by 72 °C for 10 min. PCR amplicons were cleaned and indexed as indicated in the Illumina’s MiSeq 16S Sequencing Library Protocol and sequenced with Miseq (2 × 300 pb). Sequencing was performed at the Genomics Center (FISABIO, Valencia, Spain).

### Microbiome analysis

The sequenced data was quality filtered using prinseq-lite^83^, eliminating 0.89% of the reads, with the following parameters min_length: 50, trim_qual_right: 30, trim_qual_type: mean, trim_qual_window: 20 and then joined with FLASH^84^, using default parameters producing 814,069 amplicons (Supplementary Table S1). The primers were removed with cutadapt^85^, and the cleaned merged reads were analyzed with QIIME2.2020^58^. Low quality reads were eliminated with quality-filter q-score, eliminating ≈54 merged reads/ sample. Deblur was used to trim the sequences at position 417 to remove low quality regions^86^.

Diversity was studied using the QIIME2 plugin q2-diversity for C57BL/6J-rd10 mice and male–female mice^58^. Specifically, alpha-diversity was evaluated with Pielou’s Evenness, Shannon’s Diversity index and Faith’s Phylogenetic Diversity index and compared with the no-parametric Kruskal–Wallis test. Beta-diversity was studied using PERMANOVA with the Bray-Courtis distance, Jaccard distance and weighted UniFrac and unweighted UniFrac distances. PCoAs (-p-metric seuclidean) were performed for representing beta-diversity and for all the taxonomic levels, that were previously collapsed. Taxonomy was assigned with the already pre-formatted SILVA 138 database (reproducible sequence taxonomy reference database management for the masses)^87^. The comparison between taxa’s relative abundance to find differentially abundant features was performed with ANCOM^59^. Accumulative relative abundance for unique ASV (present in C57BL/6J or rd10 mice, never both) was calculated adding the relative abundance of each unique ASV.

### Immunohistochemistry

Immunohistochemical assessment of the retinas was achieved following previously reported methodology^52^. Briefly, retinal sections were thawed at room temperature, washed 3 times with PB and incubated for 1 h in 0.1 M PB with 10% (v/v) normal donkey serum and 0.5% Triton X-100. After that, sections were immunolabeled overnight at 4 °C under agitation using combinations of primary antibodies at different dilutions in 0.1 M PB with 0.5% Triton X-100: mouse monoclonal anti-rhodopsin (MAB5356, Merk Millipore, Darmstadt, Germany, 1:100), rabbit polyclonal anti-cone arrestin (AB15282, Merk Millipore, 1:200), rabbit polyclonal anti-ionized calcium-binding adapter molecule 1 (Iba1) (019-19741, Wako Chemicals, Richmond, VA, USA, 1:1000) and mouse monoclonal anti-glial fibrillary acidic protein (GFAP) (G3893, Sigma-Aldrich, Steinheim, Germany, 1:500). For objective comparison, rd10 and C57BL/6J retinas were processed in parallel. The slides were washed and then incubated with a mixture of corresponding secondary antibodies at a dilution of 1:100 in PB with 0.5% Triton X-100: AlexaFluor 488-anti-rabbit and AlexaFluor 555-anti-mouse (Invitrogen, Carlsbad, CA, USA). When corresponded, the nuclei marker TO-PRO 3-iodide (Invitrogen) was added at a dilution of 1:1000. Images were acquired on a Leica TCS SP8 confocal laser-scanning microscope (Leica Microsystems, Wetzlar, Germany).

### Measurement of retina outer nuclear layer thickness

In order to assess photoreceptor death in retinal degenerative conditions, the thickness of the outer nuclear layer (ONL) was quantified in at least two non-consecutive sections per retina stained with hematoxylin. Retinal sections included the optic nerve and the temporal and nasal *ora serrata.* As the progression of the degeneration is not uniform throughout the retina, the quantification was performed every 0.5 mm, at distances of 0, 0.5, 1.0, 1.5, 2.0 and 2.3 mm from the optic nerve toward the periphery.

### Statistical analysis

A one-way ANOVA was performed to assess the effects of genotype (rd10 vs. C57BL/6J) on ERG amplitude and ONL thickness, using the IBM SPSS statistics 24 software package (SPSS Inc, Chicago, IL, USA). Post hoc pairwise comparisons were done with the Bonferroni’s test. To assess the effects of genotype on visual acuity, a Mann–Whitney U test was applied. Diversity parameters were statistically evaluated using different QIIME2 tools (https://qiime2.org/): the nonparametric Kruskal–Wallis test was used to compare alpha-diversity whereas beta-diversity was studied using PERMANOVA. The comparison between taxa’s relative abundance was performed with ANCOM^59^, which found features that were more abundant in a group as compared with the other. One-way ANOVA was applied to study abundance differences between different taxon levels and ASV numbers using the R statistical software (4.0.2)^88^. A p value of less than 0.05 was considered to be statistically significant. All data were plotted as the average ± standard error of the mean.

### Ethics declarations

All procedures were performed in conformity with current guidelines and regulations on the use of laboratory animals (European Directive 2010/63/EU, NIH and ARVO) in an effort to reduce the number of animals used and limit unnecessary animal suffering.

### Approval for animal experiments

This study was approved by the Ethics Committee of the University of Alicante (UA-2018–07-06).

## Supplementary Information

Supplementary Information

## Data Availability

The 16 s rRNA raw sequences generated during the current study were deposited at Sequence Read Archive (SRA) database which belongs to the National Center for Biotechnology Information. Bioproject number: PRJNA675447. Biosamples ID for C57BL/6J mice: SAMN16708365 (mouse 25), SAMN16708366 (mouse 26), SAMN16708367 (mouse 27) and SAMN16708368 (mouse 32). Biosamples ID for rd10 mice: SAMN16708371 (mouse 88), SAMN16708372 (mouse 99), SAMN16708369 (mice 102) and SAMN16708370 (mouse109).

## References

[1] Mayer EA (2011). Gut feelings: the emerging biology of gut-brain communication. Nat. Rev Neurosci.

[2] Klingelhoefer L, Reichmann H (2015). Pathogenesis of Parkinson disease–the gut-brain axis and environmental factors. Nat. Rev. Neurol..

[3] Powell N, Walker MM, Talley NJ (2017). The mucosal immune system: master regulator of bidirectional gut-brain communications. Nat. Rev. Gastroenterol. Hepatol..

[4] Cryan JF, Dinan TG (2012). Mind-altering microorganisms: the impact of the gut microbiota on brain and behaviour. Nat. Rev. Neurosci..

[5] Cryan JF (2019). The microbiota-gut-brain axis. Physiol. Rev..

[6] Sharon G, Sampson TR, Geschwind DH, Mazmanian SK (2016). The central nervous system and the gut microbiome. Cell.

[7] Torres-Fuentes C, Schellekens H, Dinan TG, Cryan JF (2017). The microbiota-gut-brain axis in obesity. Lancet Gastroenterol. Hepatol..

[8] Dalile B, Van Oudenhove L, Vervliet B, Verbeke K (2019). The role of short-chain fatty acids in microbiota-gut-brain communication. Nat. Rev. Gastroenterol. Hepatol..

[9] Qin J (2012). A metagenome-wide association study of gut microbiota in type 2 diabetes. Nature.

[10] Sudo N (2004). Postnatal microbial colonization programs the hypothalamic-pituitary-adrenal system for stress response in mice. J. Physiol..

[11] Diaz Heijtz R (2011). Normal gut microbiota modulates brain development and behavior. Proc. Natl. Acad. Sci. U. S. A..

[12] Farmer AD, Randall HA, Aziz Q (2014). It's a gut feeling: how the gut microbiota affects the state of mind. J. Physiol..

[13] Desbonnet L, Clarke G, Shanahan F, Dinan TG, Cryan JF (2014). Microbiota is essential for social development in the mouse. Mol. Psychiatry.

[14] Gao X (2018). Chronic stress promotes colitis by disturbing the gut microbiota and triggering immune system response. Proc. Natl. Acad. Sci. U. S. A..

[15] Jang SE (2018). Gastrointestinal inflammation by gut microbiota disturbance induces memory impairment in mice. Mucosal Immunol..

[16] Fung TC, Olson CA, Hsiao EY (2017). Interactions between the microbiota, immune and nervous systems in health and disease. Nat. Neurosci..

[17] Dinan TG, Cryan JF (2017). Gut instincts: microbiota as a key regulator of brain development, ageing and neurodegeneration. J. Physiol..

[18] Sherwin E, Dinan TG, Cryan JF (2018). Recent developments in understanding the role of the gut microbiota in brain health and disease. Ann. N. Y. Acad. Sci..

[19] Strandwitz P (2018). Neurotransmitter modulation by the gut microbiota. Brain Res..

[20] Fournier CN, Houser M, Tansey MG, Glass JD, Hertzberg VS (2020). The gut microbiome and neuroinflammation in amyotrophic lateral sclerosis? Emerging clinical evidence. Neurobiol. Dis..

[21] Benakis C (2016). Commensal microbiota affects ischemic stroke outcome by regulating intestinal gammadelta T cells. Nat. Med..

[22] Singh V (2016). Microbiota dysbiosis controls the neuroinflammatory response after stroke. J. Neurosci..

[23] Sundman MH, Chen NK, Subbian V, Chou YH (2017). The bidirectional gut-brain-microbiota axis as a potential nexus between traumatic brain injury, inflammation, and disease. Brain Behav. Immun..

[24] Houlden A (2016). Brain injury induces specific changes in the caecal microbiota of mice via altered autonomic activity and mucoprotein production. Brain Behav. Immun..

[25] Scheperjans F (2015). Gut microbiota are related to Parkinson's disease and clinical phenotype. Mov. Disord..

[26] Sampson TR (2016). Gut microbiota regulate motor deficits and neuroinflammation in a model of Parkinson's disease. Cell.

[27] Houser MC, Tansey MG (2017). The gut-brain axis: is intestinal inflammation a silent driver of Parkinson's disease pathogenesis?. NPJ Parkinsons Dis..

[28] Cryan JF, O'Riordan KJ, Sandhu K, Peterson V, Dinan TG (2020). The gut microbiome in neurological disorders. Lancet Neurol..

[29] Kong G (2020). Microbiome profiling reveals gut dysbiosis in a transgenic mouse model of Huntington's disease. Neurobiol. Dis..

[30] Cuenca N (2014). Cellular responses following retinal injuries and therapeutic approaches for neurodegenerative diseases. Prog. Retin. Eye Res..

[31] Ortuno-Lizaran I (2018). Phosphorylated alpha-synuclein in the retina is a biomarker of Parkinson's disease pathology severity. Mov. Disord..

[32] Veys L (2019). Retinal alpha-synuclein deposits in Parkinson's disease patients and animal models. Acta Neuropathol..

[33] Rowan S, Taylor A (2018). The role of microbiota in retinal disease. Adv. Exp. Med. Biol..

[34] Nayyar A, Gindina S, Barron A, Hu Y, Danias J (2020). Do epigenetic changes caused by commensal microbiota contribute to development of ocular disease? A review of evidence. Hum. Genomics.

[35] Zinkernagel MS (2017). Association of the intestinal microbiome with the development of neovascular age-related macular degeneration. Sci. Rep..

[36] Rowan S (2017). Involvement of a gut-retina axis in protection against dietary glycemia-induced age-related macular degeneration. Proc. Natl. Acad. Sci. U. S. A..

[37] Rinninella E (2018). The role of diet, micronutrients and the gut microbiota in age-related macular degeneration: new perspectives from the gut(-)retina axis. Nutrients.

[38] Rowan S, Taylor A (2018). Gut microbiota modify risk for dietary glycemia-induced age-related macular degeneration. Gut Microbes.

[39] Zysset-Burri DC (2020). Associations of the intestinal microbiome with the complement system in neovascular age-related macular degeneration. NPJ Genomic Med..

[40] Astafurov K (2014). Oral microbiome link to neurodegeneration in glaucoma. PLoS ONE.

[41] Gong H (2020). Gut microbiota compositional profile and serum metabolic phenotype in patients with primary open-angle glaucoma. Exp. Eye Res..

[42] Chen SD, Wang YY, Liu YM, Zhang XL (2020). Gut microbiota and related metabolomic change in primary open-angle glaucoma. Investig. Ophthalmol. Vis. Sci..

[43] Tang J, Tang Y, Yi I, Chen DF (2020). The role of commensal microflora-induced T cell responses in glaucoma neurodegeneration. Prog. Brain Res..

[44] Sisinthy S (2020). Alterations in the gut bacterial microbiome in diabetic mellitus and diabetic retinopathy patients.. Investig Ophthalmol. Vis. Sci..

[45] Maneu V (2016). Immunosuppression, peripheral inflammation and invasive infection from endogenous gut microbiota activate retinal microglia in mouse models. Microbiol. Immunol..

[46] Noailles A, Fernandez-Sanchez L, Lax P, Cuenca N (2014). Microglia activation in a model of retinal degeneration and TUDCA neuroprotective effects. J. Neuroinflamm..

[47] Noailles A (2016). Persistent inflammatory state after photoreceptor loss in an animal model of retinal degeneration. Sci. Rep..

[48] Noailles A, Maneu V, Campello L, Lax P, Cuenca N (2018). Systemic inflammation induced by lipopolysaccharide aggravates inherited retinal dystrophy. Cell Death Dis..

[49] Komeima K, Rogers BS, Lu L, Campochiaro PA (2006). Antioxidants reduce cone cell death in a model of retinitis pigmentosa. Proc. Natl. Acad. Sci. U. S. A..

[50] Moreno ML, Merida S, Bosch-Morell F, Miranda M, Villar VM (2018). Autophagy dysfunction and oxidative stress, two related mechanisms implicated in retinitis pigmentosa. Front. Physiol..

[51] Campello L (2020). New Nrf2-inducer compound ITH12674 slows the progression of retinitis pigmentosa in the mouse model rd10. Cell Physiol. Biochem..

[52] Kutsyr O (2020). Gradual increase in environmental light intensity induces oxidative stress and inflammation and accelerates retinal neurodegeneration. Investig. Ophthalmol. Vis. Sci..

[53] Huang L (2017). Mutation screening in genes known to be responsible for Retinitis Pigmentosa in 98 Small Han Chinese Families. Sci. Rep..

[54] Chang B (2002). Retinal degeneration mutants in the mouse. Vis. Res..

[55] Chang B (2007). Two mouse retinal degenerations caused by missense mutations in the beta-subunit of rod cGMP phosphodiesterase gene. Vis. Res..

[56] Wang T (2018). The PDE6 mutation in the rd10 retinal degeneration mouse model causes protein mislocalization and instability and promotes cell death through increased ion influx. J. Biol. Chem..

[57] Roche SL (2016). Progesterone attenuates microglial-driven retinal degeneration and stimulates protective fractalkine-CX3CR1 signaling. PLoS ONE.

[58] Bolyen E (2019). Reproducible, interactive, scalable and extensible microbiome data science using QIIME 2 (vol 37, pg 852, 2019). Nat. Biotechnol..

[59] Mandal S (2015). Analysis of composition of microbiomes: a novel method for studying microbial composition. Microb. Ecol. Health Dis..

[60] Callahan BJ, McMurdie PJ, Holmes SP (2017). Exact sequence variants should replace operational taxonomic units in marker-gene data analysis. ISME J..

[61] Knight R (2018). Best practices for analysing microbiomes. Nat. Rev. Microbiol..

[62] Callahan BJ (2016). DADA2: High-resolution sample inference from Illumina amplicon data. Nat. Methods.

[63] Eren AM (2013). Oligotyping: differentiating between closely related microbial taxa using 16S rRNA gene data. Methods Ecol. Evol..

[64] Eren AM (2015). Minimum entropy decomposition: unsupervised oligotyping for sensitive partitioning of high-throughput marker gene sequences. ISME J..

[65] Needham DM, Sachdeva R, Fuhrman JA (2017). Ecological dynamics and co-occurrence among marine phytoplankton, bacteria and myoviruses shows microdiversity matters. ISME J..

[66] Lozupone CA, Hamady M, Kelley ST, Knight R (2007). Quantitative and qualitative beta diversity measures lead to different insights into factors that structure microbial communities. Appl. Environ. Microbiol..

[67] Jousset A (2017). Where less may be more: how the rare biosphere pulls ecosystems strings. ISME J..

[68] Benjamino J, Lincoln S, Srivastava R, Graf J (2018). Low-abundant bacteria drive compositional changes in the gut microbiota after dietary alteration. Microbiome.

[69] Wang JJ (2019). Core gut bacteria analysis of healthy mice. Front. Microbiol..

[70] Volk JK (2019). The Nlrp6 inflammasome is not required for baseline colonic inner mucus layer formation or function. J. Exp. Med..

[71] Smith BJ (2019). Changes in the gut microbiome and fermentation products concurrent with enhanced longevity in acarbose-treated mice. BMC Microbiol..

[72] Li AL (2020). Effect of 2 '-fucosyllactose supplementation on intestinal flora in mice with intestinal inflammatory diseases. Int. Dairy J..

[73] Unger MM (2016). Short chain fatty acids and gut microbiota differ between patients with Parkinson's disease and age-matched controls. Parkinsonism Relat. Disord..

[74] Chen J (2016). Multiple sclerosis patients have a distinct gut microbiota compared to healthy controls. Sci. Rep..

[75] Jiang HY (2015). Altered fecal microbiota composition in patients with major depressive disorder. Brain Behav. Immun..

[76] Fernandez J (2016). Colon microbiota fermentation of dietary prebiotics towards short-chain fatty acids and their roles as anti-inflammatory and antitumour agents: a review. J. Funct. Foods.

[77] Gill PA, van Zelm MC, Muir JG, Gibson PR (2018). short chain fatty acids as potential therapeutic agents in human gastrointestinal and inflammatory disorders. Aliment. Pharmacol. Ther..

[78] Luca M, Di Mauro M, Perry G (2019). Neuropsychiatric disturbances and diabetes mellitus: the role of oxidative stress. Oxid. Med. Cell. Longev..

[79] Li XJ (2020). Bidirectional brain-gut-microbiota axis in increased intestinal permeability induced by central nervous system injury. CNS Neurosci. Ther..

[80] Ho EXP (2018). Human pharyngeal microbiota in age-related macular degeneration. PLoS ONE.

[81] Andriessen EM (2016). Gut microbiota influences pathological angiogenesis in obesity-driven choroidal neovascularization. EMBO Mol. Med..

[82] Beli E (2018). Restructuring of the gut microbiome by intermittent fasting prevents retinopathy and prolongs survival in db/db mice. Diabetes.

[83] Schmieder R, Edwards R (2011). Quality control and preprocessing of metagenomic datasets. Bioinformatics.

[84] Magoc T, Salzberg SL (2011). FLASH: fast length adjustment of short reads to improve genome assemblies. Bioinformatics.

[85] Martin M (2011). Cutadapt removes adapter sequences from high-throughput sequencing reads. EMBnet. J..

[86] Amir A (2017). Deblur rapidly resolves single-nucleotide community sequence patterns. mSystems.

[87] Quast C (2013). The SILVA ribosomal RNA gene database project: improved data processing and web-based tools. Nucleic Acids Res..

[88] R Core Team (2020). R: A language and environment for statistical computing.

