# Supplementary material for: Retinitis pigmentosa is associated with shifts in the gut microbiome
Source: Sci Rep. 2021 Mar 23;11:6692. doi: 10.1038/s41598-021-86052-1 (PMC7988170; doi:10.1038/s41598-021-86052-1)
Supplement: Supplementary file 1 — Supplementary Information [file 41598_2021_86052_MOESM1_ESM.pdf]

# Retinitis pigmentosa is associated with shifts in the gut microbiome

Oksana Kutsyr[1+], Lucía Maestre-Carballa[1+], Mónica Lluesma-Gomez[1], Manuel Martinez-Garcia[1*], Nicolás Cuenca[1,2], Pedro Lax[1*]

[1]Department of Physiology, Genetics and Microbiology, University of Alicante, Alicante, Spain.

[2]Institute Ramón Margalef, University of Alicante, Alicante, Spain.

Email addresses:

Oksana Kutsyr: oksana.kutsyr@ua.es

Lucía Maestre-Carballa: l.maestre@ua.es

Mónica Lluesma-Gomez: m.lluesma@ua.es

Manuel Martinez-Garcia: m.martinez@ua.es

Nicolás Cuenca: cuenca@ua.es

Pedro Lax: pedro.lax@ua.es

**Supplementary Table S1.** Number of reads variation previous to QIIME2 analysis.

| Sample name | No. Raw Sequenced reads | No. Reads after QC | No. Joined reads |
|---|---|---|---|
| **C57.25** | F: 157704 | F: 156314 | 133979 |
| | R: 157704 | R: 156314 | |
| **C57.26** | F: 120828 | F: 119283 | 94596 |
| | R: 120828 | R: 119283 | |
| **C57.27** | F: 110848 | F: 109902 | 93635 |
| | R: 110848 | R: 109902 | |
| **C57.32** | F: 115625 | F: 114685 | 98528 |
| | R: 115625 | R: 114685 | |
| **rd10.88** | F: 125328 | F: 124248 | 108651 |
| | R: 125328 | R: 124248 | |
| **rd10.89** | F: 98154 | F: 97336 | 84398 |
| | R: 98154 | R: 97336 | |
| **rd10.102** | F: 101379 | F: 100547 | 85878 |
| | R: 101379 | R: 100547 | |
| **rd10.109** | F: 130339 | F: 129309 | 114404 |
| | R: 130339 | R: 129309 | |

For the 8 samples tested (4 from C57BL/6J mice and 4 from rd10 mice): Number (No.) of reads decrease from sequenced raw reads (No. Raw Sequenced reads) to merged reads with FLASH (No. Joined reads) [1], including the passing reads of the quality filtering step performed with prinseq-lite (No. Reads after QC) [2]. F: Forward Read. R: Reverse Read.

**Supplementary Table S2**. Deblur denoising statistics.

| Sample | Reads | Filtered reads | Percentage of input passed filter | Denoised | Chimeras | No. of final Reads | % of reads that passed the denoiser | No. ASV found |
|---|---|---|---|---|---|---|---|---|
| **C57.25** | 133904 | 86027 | 64.25 | 50360 | 1408 | 48952 | 36.56 | 94 |
| **C57.26** | 94555 | 60103 | 63.56 | 33873 | 1720 | 32153 | 34.00 | 92 |
| **C57.27** | 93592 | 54585 | 58.32 | 34085 | 767 | 33318 | 35.60 | 96 |
| **C57.32** | 98477 | 55385 | 56.24 | 35569 | 320 | 35249 | 35.79 | 94 |
| **rd10.102** | 85821 | 53760 | 62.64 | 36889 | 1338 | 35551 | 41.42 | 84 |
| **rd10.109** | 114339 | 65513 | 57.30 | 42684 | 708 | 41976 | 36.71 | 88 |
| **rd10.88** | 108599 | 76453 | 70.40 | 48081 | 1771 | 46310 | 42.64 | 83 |
| **rd10.89** | 84350 | 51617 | 61.19 | 35118 | 2151 | 32967 | 39.08 | 88 |

Deblur [3] denoiser' s effect in different samples (C57BL/6J and rd10 mice). The input (Reads input) was filtered, removing singletons and artifacts (Filtered reads passed). Then, reads were denoised (Denoised), the chimeras were found and eliminated (Chimeras), to produce the final reads count (No. of final Reads), and the % of the final reads compared with the initial input (% of reads that passed the denoiser). Last column shows the number of ASV found in each sample (No. ASV found).

**Supplementary Table S3.** Unique ASV and relative abundance.

| SILVA 138 Classification | ASV ID | Relative abundance c57 (%) | Relative abundance rd10 (%) |
|---|---|---|---|
| **d__Bacteria; p__Bacteroidota; c__Bacteroidia; o__Bacteroidales; f__Muribaculaceae; g__Muribaculaceae; s__uncultured_bacterium** | 8fe55c3953a92b957f64876d03aed609 | 0 | 6,85323601 |
| **d__Bacteria; p__Bacteroidota; c__Bacteroidia; o__Bacteroidales; f__Muribaculaceae; g__Muribaculaceae** | 4b439e9677e560cbf2178aa2a0c1be47 | 0 | 2,36829362 |
| **d__Bacteria; p__Bacteroidota; c__Bacteroidia; o__Bacteroidales; f__Rikenellaceae; g__Alistipes; s__uncultured_bacterium** | f12afab62c73afc04a7671678ef9de96 | 0 | 1,68562908 |
| **d__Bacteria; p__Bacteroidota; c__Bacteroidia; o__Bacteroidales; f__Muribaculaceae; g__Muribaculaceae; s__uncultured_Bacteroidales** | a5d25160ed7029c3aaa6a1a68372f63f | 0 | 0,83425545 |
| **d__Bacteria; p__Bacteroidota; c__Bacteroidia; o__Bacteroidales; f__Bacteroidaceae; g__Bacteroides; s__Bacteroides_caecimuris** | 4a178a9e45c6faf13bdadbd53a4f2028 | 0 | 0,70306996 |
| **d__Bacteria; p__Bacteroidota; c__Bacteroidia; o__Bacteroidales; f__Marinifilaceae; g__Odoribacter** | 003eb23d0c6f66f14a53fdd762abe6e9 | 0 | 0,69504002 |
| **d__Bacteria; p__Bacteroidota; c__Bacteroidia; o__Bacteroidales; f__Muribaculaceae; g__Muribaculaceae** | 32e289e3b1914406c8f7fc140c216d61 | 0 | 0,50655507 |
| **d__Bacteria; p__Bacteroidota; c__Bacteroidia; o__Bacteroidales; f__Muribaculaceae; g__Muribaculaceae; s__uncultured_bacterium** | dc9f79ac9e6f880da114f520a216e45e | 0 | 0,4446846 |
| **d__Bacteria; p__Bacteroidota; c__Bacteroidia; o__Bacteroidales; f__Bacteroidaceae; g__Bacteroides** | 515255b61da63b9e602b78270f3f4dad | 0 | 0,39556033 |
| **d__Bacteria; p__Bacteroidota; c__Bacteroidia; o__Bacteroidales; f__Muribaculaceae; g__Muribaculaceae; s__uncultured_Bacteroidales** | 5784fa5805f604f9472e5676e2f881c7 | 0 | 0,3314337 |
| **d__Bacteria; p__Bacteroidota; c__Bacteroidia; o__Bacteroidales; f__Rikenellaceae; g__Alistipes; s__uncultured_bacterium** | efe3ee8ed5ea37809f29b5943742dfed | 0 | 0,29768771 |
| **d__Bacteria; p__Bacteroidota; c__Bacteroidia; o__Bacteroidales; f__Rikenellaceae; g__Alistipes; s__uncultured_bacterium** | 302a42730df2ccd3c661841b6421f30a | 0 | 0,28187409 |
| **d__Bacteria; p__Bacteroidota; c__Bacteroidia; o__Bacteroidales; f__Bacteroidaceae; g__Bacteroides** | c3a6d64ff6f5672832fa1393194ac562 | 0 | 0,26233929 |
| **d__Bacteria; p__Bacteroidota; c__Bacteroidia; o__Bacteroidales; f__Muribaculaceae; g__Muribaculaceae; s__uncultured_bacterium** | d4847c37d6e999e9f1398b34d26e8b92 | 0 | 0,25075174 |
| **d__Bacteria; p__Firmicutes; c__Clostridia; o__Lachnospirales; f__Lachnospiraceae; g__Lachnospiraceae_NK4A136_group** | 0e370fd4ec92f50c541e5572b562a397 | 0 | 0,19086114 |

| | | | |
|---|---|---|---|
| **d__Bacteria; p__Bacteroidota; c__Bacteroidia; o__Bacteroidales; f__Muribaculaceae; g__Muribaculaceae** | d17d221a71fcfb3cf0e3d5a13438ee2f | 0 | 0,17222883 |
| **d__Bacteria; p__Bacteroidota; c__Bacteroidia; o__Bacteroidales; f__Muribaculaceae; g__Muribaculaceae; s__uncultured_Bacteroidales** | 0d293735d22331898a4413a8d43a8806 | 0 | 0,16187567 |
| **d__Bacteria; p__Firmicutes; c__Bacilli; o__Staphylococcales; f__Staphylococcaceae; g__Staphylococcus** | f79a13aeeb02307eac37dae9c73fb26a | 0 | 0,11256413 |
| **d__Bacteria; p__Firmicutes; c__Bacilli; o__Lactobacillales; f__Enterococcaceae; g__Enterococcus** | 1a5b347c9d6b40bff9b504d7126e5b19 | 0 | 0,08910462 |
| **d__Bacteria; p__Bacteroidota; c__Bacteroidia; o__Bacteroidales; f__Muribaculaceae; g__Muribaculaceae; s__uncultured_bacterium** | 2590da681572248f1046446ebb810538 | 0 | 0,08670496 |
| **d__Bacteria; p__Bacteroidota; c__Bacteroidia; o__Bacteroidales; f__Muribaculaceae; g__Muribaculaceae** | e33516650520a532b7f62148db647207 | 0 | 0,08629969 |
| **d__Bacteria; p__Bacteroidota; c__Bacteroidia; o__Bacteroidales; f__Muribaculaceae; g__Muribaculaceae; s__uncultured_Bacteroidales** | 5487a84271b262185804ed07cd015753 | 0 | 0,07758391 |
| **d__Bacteria; p__Bacteroidota; c__Bacteroidia; o__Bacteroidales; f__Prevotellaceae; g__Prevotellaceae_UCG-001; s__uncultured_bacterium** | c7a5584ad3e77067f7c1587fa16e2037 | 0 | 0,07588774 |
| **d__Bacteria; p__Bacteroidota; c__Bacteroidia; o__Bacteroidales; f__Muribaculaceae; g__Muribaculaceae; s__uncultured_bacterium** | 8708905fa4617d0071a59b7829baa4e2 | 0 | 0,07043285 |
| **d__Bacteria; p__Bacteroidota; c__Bacteroidia; o__Bacteroidales; f__Muribaculaceae; g__Muribaculaceae; s__uncultured_bacterium** | a6e24cf4ebe57d3b2d30de7fd3182f7c | 0 | 0,06440346 |
| **d__Bacteria; p__Bacteroidota; c__Bacteroidia; o__Bacteroidales; f__Muribaculaceae; g__Muribaculaceae; s__uncultured_bacterium** | dfda03b9b3de00a1929a737fb4ebeafa | 0 | 0,06316064 |
| **d__Bacteria; p__Bacteroidota; c__Bacteroidia; o__Bacteroidales; f__Marinifilaceae; g__Odoribacter; s__uncultured_bacterium** | dfa994441b74f436d590e3e612929930 | 0 | 0,060749 |
| **d__Bacteria; p__Bacteroidota; c__Bacteroidia; o__Bacteroidales; f__Muribaculaceae; g__Muribaculaceae; s__uncultured_bacterium** | 6651d3d542a93c649aa39448ca8ee015 | 0 | 0,05657995 |
| **d__Bacteria; p__Bacteroidota; c__Bacteroidia; o__Bacteroidales; f__Rikenellaceae; g__Alistipes; s__uncultured_bacterium** | b0c501d54eae1676b5820a1238051360 | 0 | 0,04861066 |
| **d__Bacteria; p__Bacteroidota; c__Bacteroidia; o__Bacteroidales; f__Muribaculaceae; g__Muribaculaceae; s__uncultured_bacterium** | 54ddc8458d991fce45d039ea11be41ec | 0 | 0,04149265 |
| **d__Bacteria; p__Bacteroidota; c__Bacteroidia; o__Bacteroidales; f__Muribaculaceae; g__Muribaculaceae; s__uncultured_bacterium** | 34a77af51d3dbc2e93ef09af2805ce8c | 0 | 0,03972629 |

| | | | |
|---|---|---|---|
| **d__Bacteria; p__Firmicutes; c__Clostridia; o__Lachnospirales; f__Lachnospiraceae; g__Lachnospiraceae_NK4A136_group; s__uncultured_bacterium** | 73612fb29191406b37ff36b0db4d44ba | 0 | 0,03156566 |
| **d__Bacteria; p__Bacteroidota; c__Bacteroidia; o__Bacteroidales; f__Muribaculaceae; g__Muribaculaceae; s__uncultured_bacterium** | 69494f3650ae0fd8a0319c72b9aca342 | 0 | 0,02348169 |
| **d__Bacteria; p__Bacteroidota; c__Bacteroidia; o__Bacteroidales; f__Rikenellaceae; g__Alistipes; s__uncultured_bacterium** | 0e815c6449e618b4da0fce6a96c6aad7 | 0 | 0,0220364 |
| **d__Bacteria; p__Bacteroidota; c__Bacteroidia; o__Bacteroidales; f__Muribaculaceae; g__Muribaculaceae; s__uncultured_Bacteroidales** | fb89b85997d260628c126c5718b84635 | 0 | 0,02105411 |
| **d__Bacteria; p__Firmicutes; c__Clostridia; o__Lachnospirales; f__Lachnospiraceae** | 01f5b34acc5a84f0b40314f6416bcb76 | 0 | 0,01608062 |
| **d__Bacteria; p__Firmicutes; c__Clostridia; o__Lachnospirales; f__Lachnospiraceae; g__Lachnospiraceae_NK4A136_group** | f97ebde97cd04fdc3a882088b76f0ca6 | 0 | 0,01310273 |
| **d__Bacteria; p__Bacteroidota; c__Bacteroidia; o__Bacteroidales; f__Muribaculaceae; g__Muribaculaceae** | c862d7e33284897b9583e48965f02f51 | 0 | 0,01265787 |
| **d__Bacteria; p__Firmicutes; c__Bacilli; o__Staphylococcales; f__Staphylococcaceae; g__Jeotgalicoccus** | 61846f6550679f1eef066723da13f7c8 | 0 | 0,01223422 |
| **d__Bacteria; p__Bacteroidota; c__Bacteroidia; o__Bacteroidales; f__Muribaculaceae; g__Muribaculaceae** | 5ea89ed231e3db9b2f5d4b718660ae6d | 0 | 0,01012483 |
| **d__Bacteria; p__Bacteroidota; c__Bacteroidia; o__Bacteroidales; f__Muribaculaceae; g__Muribaculaceae** | 4990c0d22cc0fde25bacb3429d9a3f52 | 0 | 0,00984501 |
| **d__Bacteria; p__Firmicutes; c__Bacilli; o__Erysipelotrichales; f__Erysipelotrichaceae; g__Turicibacter; s__uncultured_bacterium** | f8e53fb45dd0bf3f6e053cedc4c207ae | 0 | 0,00984501 |
| **d__Bacteria; p__Firmicutes; c__Clostridia** | b37e76a9cf11e9d60457135e84e5713e | 0 | 0,00952421 |
| **d__Bacteria; p__Bacteroidota; c__Bacteroidia; o__Bacteroidales; f__Bacteroidaceae; g__Bacteroides** | 9325c913e0755dd75ac1271ca5d7b7f6 | 0 | 0,00875308 |
| **d__Bacteria; p__Bacteroidota; c__Bacteroidia; o__Bacteroidales; f__Rikenellaceae; g__Alistipes; s__uncultured_bacterium** | ca913340b37d7733139efb464c47fb21 | 0 | 0,00774252 |
| **d__Bacteria; p__Firmicutes; c__Clostridia; o__Lachnospirales; f__Lachnospiraceae** | 8cb99e773f716c1342f9cfbc29479a0a | 0 | 0,00665226 |
| **d__Bacteria; p__Firmicutes; c__Clostridia; o__Lachnospirales; f__Lachnospiraceae** | 584ef1f217e3bc89457c06a00d048107 | 0 | 0,00655136 |
| **d__Bacteria; p__Firmicutes; c__Bacilli; o__Erysipelotrichales; f__Erysipelotrichaceae; g__uncultured; s__unidentified** | 3188c1bdeddaeb3a625004b41b01ff4d | 0 | 0,00627238 |
| **d__Bacteria; p__Firmicutes; c__Clostridia; o__Lachnospirales; f__Lachnospiraceae** | 74c52127c1bea3f1f94c58f21ca00558 | 0 | 0,00605388 |

| | | | |
|---|---|---|---|
| **d__Bacteria; p__Bacteroidota; c__Bacteroidia; o__Bacteroidales; f__Muribaculaceae; g__Muribaculaceae; s__uncultured_bacterium** | 452433a28f3bbc3f70da063824cd6bbf | 0,00777532 | 0 |
| **d__Bacteria; p__Firmicutes; c__Clostridia; o__Peptococcales; f__Peptococcaceae; g__uncultured; s__uncultured_bacterium** | 861c32723edae1ab3abb31f7e3469514 | 0,00801774 | 0 |
| **d__Bacteria; p__Firmicutes; c__Clostridia; o__Peptococcales; f__Peptococcaceae; g__uncultured; s__unidentified** | 0a8dc3b5bb8df0eb1834f3aed9744e95 | 0,00804827 | 0 |
| **d__Bacteria; p__Firmicutes; c__Clostridia; o__Lachnospirales; f__Lachnospiraceae; g__Lachnospiraceae_NK4A136_group** | 2bd246c6114e43805cd8c6c6320b8991 | 0,00817127 | 0 |
| **d__Bacteria; p__Bacteroidota; c__Bacteroidia; o__Bacteroidales; f__Muribaculaceae; g__Muribaculaceae; s__uncultured_Bacteroidales** | 81a5a5e5b7fe8650536683ab8c439c2d | 0,01059063 | 0 |
| **d__Bacteria; p__Firmicutes; c__Bacilli; o__RF39; f__RF39; g__RF39; s__uncultured_bacterium** | df8a6e092c5a3d2f3993c34108287999 | 0,0106386 | 0 |
| **d__Bacteria; p__Bacteroidota; c__Bacteroidia; o__Bacteroidales; f__Muribaculaceae; g__Muribaculaceae; s__uncultured_Bacteroidales** | a0009e240781ae79fffd5f9e560b96b3 | 0,01260895 | 0 |
| **d__Bacteria; p__Bacteroidota; c__Bacteroidia; o__Bacteroidales; f__Muribaculaceae; g__Muribaculaceae; s__uncultured_Bacteroidales** | bf4c38add9b7b73e53d4a0ecfb85900a | 0,01450331 | 0 |
| **d__Bacteria; p__Bacteroidota; c__Bacteroidia; o__Bacteroidales; f__Muribaculaceae; g__Muribaculaceae** | da77008241881cd622c6e32564cf1293 | 0,01552346 | 0 |
| **d__Bacteria; p__Bacteroidota; c__Bacteroidia; o__Bacteroidales; f__Muribaculaceae; g__Muribaculum; s__uncultured_bacterium** | 76ba7d55c7fa461d33b29f315b266735 | 0,01684689 | 0 |
| **d__Bacteria; p__Bacteroidota; c__Bacteroidia; o__Bacteroidales; f__Muribaculaceae; g__Muribaculaceae; s__uncultured_bacterium** | 76339fd65b9741cc235e74469b7505b6 | 0,02029581 | 0 |
| **d__Bacteria; p__Bacteroidota; c__Bacteroidia; o__Bacteroidales; f__Muribaculaceae; g__Muribaculaceae; s__uncultured_Bacteroidales** | a384a57add3a050f64b65123e6b29e4c | 0,02136339 | 0 |
| **d__Bacteria; p__Bacteroidota; c__Bacteroidia; o__Bacteroidales; f__Muribaculaceae; g__Muribaculaceae** | 83a1199ece86f770179563e69aefc4ff | 0,02864535 | 0 |
| **d__Bacteria; p__Bacteroidota; c__Bacteroidia; o__Bacteroidales; f__Muribaculaceae; g__Muribaculaceae; s__uncultured_organism** | 50f8c7d97bfbb7e7ab445a43ff7d5497 | 0,03049732 | 0 |
| **d__Bacteria; p__Bacteroidota; c__Bacteroidia; o__Bacteroidales; f__Muribaculaceae; g__Muribaculaceae; s__uncultured_Bacteroidales** | 7cd293b474a6426e4d38d421c71df82e | 0,03226484 | 0 |
| **d__Bacteria; p__Bacteroidota; c__Bacteroidia; o__Bacteroidales; f__Rikenellaceae; g__Rikenellaceae_RC9_gut_group; s__uncultured_bacterium** | 929d38142d421d776e3afd228d991b39 | 0,03504928 | 0 |

| | | | |
|---|---|---|---|
| **d__Bacteria; p__Bacteroidota; c__Bacteroidia; o__Bacteroidales; f__Muribaculaceae; g__Muribaculaceae; s__uncultured_Bacteroidales** | 076b08feb8624fffb494c56458b6fdca | 0,04915723 | 0 |
| **d__Bacteria; p__Bacteroidota; c__Bacteroidia; o__Bacteroidales; f__Muribaculaceae; g__Muribaculaceae; s__uncultured_bacterium** | 81b15c3e47c2a9c6a8a8ddfb3b944ed0 | 0,04951662 | 0 |
| **d__Bacteria; p__Bacteroidota; c__Bacteroidia; o__Bacteroidales; f__Rikenellaceae; g__Alistipes** | 02666e3c9fa9366c506317bc991fb8e5 | 0,05801671 | 0 |
| **d__Bacteria; p__Firmicutes; c__Bacilli; o__RF39; f__RF39; g__RF39; s__uncultured_bacterium** | e7b8dd4833a3dc4239ecc60f22f626c9 | 0,07305172 | 0 |
| **d__Bacteria; p__Bacteroidota; c__Bacteroidia; o__Bacteroidales; f__Muribaculaceae; g__Muribaculaceae; s__uncultured_bacterium** | 6ab2d861718ae223299224694dc3856b | 0,07381994 | 0 |
| **d__Bacteria; p__Bacteroidota; c__Bacteroidia; o__Bacteroidales; f__Muribaculaceae; g__Muribaculaceae; s__uncultured_bacterium** | da9466b3aa7756ca9863c9489b9c6e99 | 0,07671257 | 0 |
| **d__Bacteria; p__Bacteroidota; c__Bacteroidia; o__Bacteroidales; f__Muribaculaceae; g__Muribaculaceae** | fb7fccd1e895b8aa572a0c4861ab7439 | 0,08634445 | 0 |
| **d__Bacteria; p__Bacteroidota; c__Bacteroidia; o__Bacteroidales; f__Rikenellaceae; g__Rikenellaceae_RC9_gut_group; s__uncultured_bacterium** | 4a1da5d3a694fe63856ba961f0a182ba | 0,08720079 | 0 |
| **d__Bacteria; p__Bacteroidota; c__Bacteroidia; o__Bacteroidales; f__Prevotellaceae; g__Prevotellaceae_UCG-001** | 1fa14fcc39b443db34eff353fe83b2eb | 0,10360187 | 0 |
| **d__Bacteria; p__Bacteroidota; c__Bacteroidia; o__Bacteroidales; f__Muribaculaceae; g__Muribaculaceae** | 6f85c2ca4faded8df244da4248136875 | 0,1313626 | 0 |
| **d__Bacteria; p__Bacteroidota; c__Bacteroidia; o__Bacteroidales; f__Rikenellaceae; g__Alistipes; s__uncultured_bacterium** | f1408d3533904b2a644333acca09958b | 0,13512363 | 0 |
| **d__Bacteria; p__Bacteroidota; c__Bacteroidia; o__Bacteroidales; f__Rikenellaceae; g__Rikenella; s__uncultured_bacterium** | f4f26450a111ac933922fe1a9c957828 | 0,14214778 | 0 |
| **d__Bacteria; p__Bacteroidota; c__Bacteroidia; o__Bacteroidales; f__Muribaculaceae; g__Muribaculaceae; s__uncultured_Bacteroidales** | 4ba388633b78983aa34359843ff8d1cf | 0,15428123 | 0 |
| **d__Bacteria; p__Bacteroidota; c__Bacteroidia; o__Bacteroidales; f__Muribaculaceae; g__Muribaculaceae; s__uncultured_bacterium** | 3ab77c93cd92d2758958fc5ce7c3c304 | 0,17732632 | 0 |
| **d__Bacteria; p__Bacteroidota; c__Bacteroidia; o__Bacteroidales; f__Muribaculaceae; g__Muribaculaceae; s__uncultured_bacterium** | 0a9b8d560680dfd0a1bf6c3efb7dee9c | 0,28469178 | 0 |
| **d__Bacteria; p__Bacteroidota; c__Bacteroidia; o__Bacteroidales; f__Muribaculaceae; g__Muribaculaceae; s__uncultured_Muribaculaceae** | 2257f0fb4e5d8fb95c6bda219b3a54aa | 0,31885831 | 0 |
| **d__Bacteria; p__Firmicutes; c__Bacilli** | e341c27da09ab0bc4406b23af9559799 | 0,43478408 | 0 |

| | | | |
|---|---|---|---|
| **d__Bacteria; p__Bacteroidota; c__Bacteroidia; o__Bacteroidales; f__Muribaculaceae; g__Muribaculaceae** | 64632422c9c6f49984eff1a2958ac858 | 0,45959887 | 0 |
| **d__Bacteria; p__Bacteroidota; c__Bacteroidia; o__Bacteroidales; f__Muribaculaceae; g__Muribaculaceae** | 654076a2db1c205e3b1b1a21f38cad2d | 0,52612402 | 0 |
| **d__Bacteria; p__Bacteroidota; c__Bacteroidia; o__Bacteroidales; f__Rikenellaceae; g__Alistipes; s__uncultured_bacterium** | 48b788d41dd55b90e90393a5a9e04f7d | 0,53584928 | 0 |
| **d__Bacteria; p__Firmicutes; c__Clostridia; o__Lachnospirales; f__Lachnospiraceae; g__Lachnospiraceae_NK4A136_group** | fce2ee99b4cb5ecdcc288c72830bd9de | 0,6197057 | 0 |
| **d__Bacteria; p__Bacteroidota; c__Bacteroidia; o__Bacteroidales; f__Rikenellaceae; g__Alistipes; s__uncultured_bacterium** | 8084921b74f294415c19175a495cf1e5 | 0,65009482 | 0 |
| **d__Bacteria; p__Bacteroidota; c__Bacteroidia; o__Bacteroidales; f__Muribaculaceae; g__Muribaculaceae; s__uncultured_Bacteroidales** | 4d5291d2b9492417101f2b90099cd2d5 | 0,76456554 | 0 |
| **d__Bacteria; p__Bacteroidota; c__Bacteroidia; o__Bacteroidales; f__Muribaculaceae; g__Muribaculaceae; s__uncultured_bacterium** | fe0a96f04a2f988c362e909a22875e99 | 0,83830256 | 0 |
| **d__Bacteria; p__Bacteroidota; c__Bacteroidia; o__Bacteroidales; f__Bacteroidaceae; g__Bacteroides** | 6cc7cfde091344267009e57804b96434 | 0,90672874 | 0 |
| **d__Bacteria; p__Firmicutes; c__Bacilli; o__Lactobacillales; f__Lactobacillaceae; g__Lactobacillus** | 5385725ae7329489f770fff4eb68c53c | 1,44174035 | 0 |
| **d__Bacteria; p__Bacteroidota; c__Bacteroidia; o__Bacteroidales; f__Muribaculaceae; g__Muribaculaceae; s__uncultured_Bacteroidales** | 02c5beb524a661ed23808ed136a98fc0 | 1,63564282 | 0 |
| **d__Bacteria; p__Bacteroidota; c__Bacteroidia; o__Bacteroidales; f__Muribaculaceae; g__Muribaculaceae; s__uncultured_bacterium** | f998eb3e30406a3e36e3ec7bbaac0aa7 | 1,6985594 | 0 |
| **d__Bacteria; p__Bacteroidota; c__Bacteroidia; o__Bacteroidales; f__Muribaculaceae; g__Muribaculaceae** | be77e43e8197e57897bb796d1c06446f | 2,17166811 | 0 |
| **d__Bacteria; p__Firmicutes; c__Clostridia; o__Lachnospirales; f__Lachnospiraceae; g__Lachnospiraceae_NK4A136_group** | 82d62f5551512bf9e6aadc2dcbaa6d4f | 2,36896612 | 0 |
| **d__Bacteria; p__Bacteroidota; c__Bacteroidia; o__Bacteroidales; f__Bacteroidaceae; g__Bacteroides** | 00db9171fb73c28da6eed2b0dd84ada2 | 2,65169135 | 0 |
| **d__Bacteria; p__Bacteroidota; c__Bacteroidia; o__Bacteroidales; f__Muribaculaceae; g__Muribaculaceae; s__uncultured_bacterium** | 32cf014be175782af90bbba43cc93bf0 | 6,71337177 | 0 |

Relative abundance (%) of unique ASVs that were found only in rd10 or C57BL/6J mice group. For each ASV ID, it is shown the SILVA 138 classification and the relative abundance (%) for the two studied mice groups.

**Supplementary Table S4.** Mice's gut microbiota alpha and beta diversity study.

| | β-diversity Distance (P) | | | | | | α-diversity Index (P) |
|---|---|---|---|---|---|---|---|
| **Samples** | **Weigthed Unifrac Distance** | **Unweighted Unifrac Distance** | **Jaccard Distance** | **Bray-courtis Distance** | **Pielou's Eveness Index** | **Shannon's Diversity Index** | **Faith's Phylogenetic Diversity Index** |
| **C57-rd10** | 0.25 | 0.03* | 0.03* | 0.03* | 0.08 | 0.08 | 0.25 |
| **Sex** | 0.31 | 0.71 | 0.62 | 0.1 | 0.25 | 0.24 | 0.56 |

Statistical p-value results (P) from the comparison of mice gut microbiota. The contrast was performed between C57BL/6J and rd10 mice (C57-rd10) or between the male and female mice (Sex). For testing the β-diversity, a PERMANOVA test was employed, while for testing the α-diversity the no-parametric Kruskal-Wallis test was used; $*p < 0.05$.

**Supplementary Table S5.** Significantly represented species according to ANCOM.

| | Deblur (W-value) | More abundant |
|---|---|---|
| ***Rikenella* spp.** | 22 | C57 |
| ***Muribaculaceace* spp.** | 27 | C57 |
| ***Prevotellaceae* UCG-001 spp.** | 23 | C57 |
| ***Bacilli* spp.** | 19 | C57 |
| ***Bacteroides caecimuris*** | 25 | rd10 |

ANCOM stat (W-value) [4] for species that were found to be statistically more abundant in control (C57) or rd10 mice (More abundant). ANCOM compares the relative abundance of each taxon with all the remaining features of the same category, showing which ones are significantly more abundant.

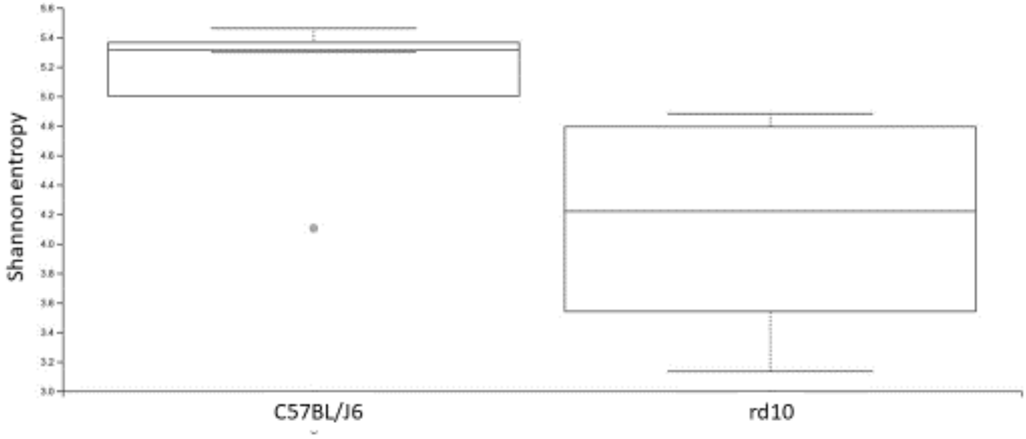


**Supplementary Figure S1.** Alpha diversity comparison between C57BL/J6 and rd10 mice gut microbiota measured with Shannon's Diversity Index and compared with no-parametric statistical Kruskal-Wallis test. No significant differences were found ($p > 0.05$).

## References


1 Magoc, T. & Salzberg, S. L. FLASH: fast length adjustment of short reads to improve genome assemblies. *Bioinformatics* **27**, 2957-2963, doi:10.1093/bioinformatics/btr507 (2011).

2 Schmieder, R. & Edwards, R. Quality control and preprocessing of metagenomic datasets. *Bioinformatics* **27**, 863-864, doi:10.1093/bioinformatics/btr026 (2011).

3 Amir, A. *et al.* Deblur Rapidly Resolves Single-Nucleotide Community Sequence Patterns. *mSystems* **2**, doi:10.1128/mSystems.00191-16 (2017).

4 Mandal, S. *et al.* Analysis of composition of microbiomes: a novel method for studying microbial composition. *Microb Ecol Health Dis* **26**, 27663, doi:10.3402/mehd.v26.27663 (2015).
